# Reviewed article: Araújo LBS, Sousa RA, Aguiar BGA, Mendonça VJ, Araújo OD, Tajra FS. Spatial and temporal distribution of neglected tropical diseases: overlap analysis, Piauí, 2013-2022. Epidemiol Serv Saude. 2026:35;e20250373

**DOI:** 10.1590/S2237-96222026v35e20250373.c

**Published:** 2026-03-16

**Authors:** Ariela Ferreira

**Affiliations:** 1 Universidade Estadual de Montes Claros, Montes Claros, MG, Brazil

## Peer review C

## Questionnaire

Does the manuscript contain new and significant information to justify publication? Yes

Does the Abstract (Summary) clearly and accurately describe the content of the article? Yes

Is the problem significant and concisely stated? Yes

Are the methods described comprehensively? Yes

Are the interpretations and conclusions justified by the results? No

Is adequate reference made to other work in the field? No

## Manuscript Structure

Length of article is: Adequate

Number of tables is: Adequate

Number of figures is: Adequate

Please state any conflict(s) of interest that you have in relation to the review of this paper. None

Interest: Good

Quality: Below Average

Originality: Average

Overall: Average

Recommendation: Major Revision

## Comments

Introdução

Após o objetivo, os autores discorrem sobre métodos e chegam a concluir sobre a contribuição do estudo.

Acredito que essa seção não poderia conter essas informações. A introdução é para conceituar e problematizar a temática.

Resumo

“Visualizou-se uma alta carga, distribuição e sobreposição desigual das doenças tropicais negligenciadas. Faz-se mais necessária a implementação de políticas públicas integrais e intersetoriais para melhoria das condições de vida e redução das desigualdades de saúde no Piauí.”

Ao falar alta carga, é juízo de valor, pois comparado a que é alta?

A sugestão é para melhorar condições de vida, mas isso não faz parte do estudo

Resultados

Não dizer: “Foram identificados 24.234 casos de Doenças Tropicais Negligenciadas no Piauí”. Afinal, não foram analisadas todas elas. Ao longo de todo o artigo, incluindo figuras, isso deve ser revisto

“Além disso, foi verificado a doença de Chagas, diferentemente das outras doenças tropicais negligenciadas”,-

Corrigir a concordância da frase

A doença de chagas avaliada foi na forma crônica ou aguda? Pois ambas são de notificação compulsória.

Além disso, citar o motivo de não ter a incidência

Quando fala-se: t”iveram alta média de prevalência”, seria alta da média de prevalência, ou a prevalência classificada como alta? Deixar mais claro no texto e incluir a classificação na figura, se for o caso.

A figura 4, nomeou as figuras como 5A e 5B- corrigir

Discussão

A discussão está superficial, e não discute com a realidade dos dados encontrados. Faltou confrontar com a literatura de forma profunda. Sugiro refazer para que os resultados relevantes sejam discutidos e confrontados com a literatura condizente. Então, abaixo deixo algumas sugestões:

-“A análise dos dados revelou um cenário preocupante em relação às doenças tropicais negligenciadas no Piauí, evidenciando não apenas sua alta prevalência, mas também a distribuição desigual entre as regiões de saúde.”

É juízo de valor, pois nem todas as doenças avaliadas tiveram alta histórica

Sugiro limitar a fazer um consolidado dos dados encontrados.

“-Observou-se que a concentração desses agravos está diretamente associada a fatores sociais, econômicos, estruturais, e não apenas biológicos, refletindo desigualdades no acesso à saúde e na implementação de estratégias de controle”

Isso não foi analisado no artigo para ter essa conclusão

-“Além disso, a sobreposição de doenças negligenciadas em determinadas áreas indica um cenário de vulnerabilidade ampliada, onde as condições de vida precárias e a limitada cobertura dos serviços de saúde favorecem a manutenção dessas enfermidades”

Isso pode ser apenas sugerido, pois não foi avaliado as condições de vida do acometidos

“-Esse padrão pode estar associado à maior exposição ocupacional, comportamentos de risco e menor busca por assistência à saúde comparado às mulheres.”

Não está referenciado, e no mesmo paragrafo traz dados com uma única referência, e além de falar do sexo, idade, fala de uma região. A referência apresentada não consegue ser o pilar de todo o parágrafo com tantos dados.

No paragrafo seguinte fala sobre melhora dos indicadores de hanseníase e tuberculose. E na discussão fala apenas da relação contextual da doença

A discussão sobre doença de chagas está incipiente. O aumento do indicador pode estar relacionado com a obrigatoriedade da notificação da forma crônica no ano de 2018. Além disso, a prevalência no estado precisa ser analisada em estudos recentes

A discussão fala de sobrecarga dos serviços de referência, mas isso não foi testado e nem está referenciado.

A discussão não levanta as limitações do estudo

